# CPM-XNet: Annotation-Efficient Deep-Learning Framework for Detecting Tuberculosis in Chest X-Ray Images

**DOI:** 10.3390/diagnostics16131947

**Published:** 2026-06-23

**Authors:** Tzu-Chin Yang, Bing-Yen Wang, Jin-Yu Li, Yu-Kang Chang, Shih-Huan Lin, Chi-Chang Chang, Yen-Wei Chu

**Affiliations:** 1 Department of Medical Imaging, Tungs’ Taichung MetroHarbor Hospital, Taichung 435403, Taiwan; tchin.yang@gmail.com; 2 Doctoral Program in Medical Biotechnology, National Chung Hsing University, Taichung City 40227, Taiwan; 3 Division of Thoracic Surgery, Department of Surgery, Changhua Christian Hospital, No. 135, Nanhsiao Street, Changhua County 500209, Taiwan; 156283@cch.org.tw; 4 Department of Post-Baccalaureate Medicine, College of Medicine, National Chung Hsing University, No. 145, Xingda Road, South Dist., Taichung City 402202, Taiwan; 5 Executive Master Program in Life Sciences, National Chung Hsing University, No. 145, Xingda Road, South Dist., Taichung City 402202, Taiwan; jerrylee@htes.tp.edu.tw; 6 Department of Medical Research, Tungs’ Taichung MetroHarbor Hospital, Taichung 435403, Taiwan; t12193@ms3.sltung.com.tw (Y.-K.C.); t15886@ms3.sltung.com.tw (S.-H.L.); 7 School of Medical Informatics, Chung Shan Medical University and IT Office, Chung Shan Medical University Hospital, Taichung 40201, Taiwan; 8 Department of Information Management, Ming Chuan University, Taoyuan 33348, Taiwan; 9 Graduate Institute of Genomics and Bioinformatics, National Chung Hsing University, Taichung City 40227, Taiwan; 10 Institute of Molecular Biology, National Chung Hsing University, Taichung City 40227, Taiwan

**Keywords:** tuberculosis, chest X-ray, deep learning, weakly supervised learning, lung-aware feature modulation, convolutional neural network, explainable AI

## Abstract

**Background/Objectives**: Chest X-ray (CXR) images are a widely used first-line screening tool for pulmonary tuberculosis (TB) detection but are difficult to interpret, which has increased demand for an automated screening tool. Deep-learning-based computer-aided diagnosis systems have demonstrated a classification performance comparable to that of trained radiologists, but they rely on dense annotations such as lesion-level or pixel-level labels, which are costly and difficult to obtain in routine clinical workflows. We developed CPM-XNet, an annotation-efficient framework for lesion-annotation-free downstream TB classification in CXR images. **Methods**: CPM-XNet incorporates a compressing–projecting mask (CPM) to provide soft lung-aware modulation while preserving global contextual information. The CPM-modulated images are then used for downstream classification with multiple convolutional neural network backbones and a vision transformer baseline. **Results**: Experiments were conducted using an internal hospital dataset and public TB datasets, and CPM-XNet showed improved performance compared with baseline models trained on unmodulated images. In a repeated-seed evaluation of the main ResNet-101 configuration on the Tung cohort, CPM-ResNet101 showed higher and more stable performance than the non-CPM counterpart and demonstrated significant paired improvement using McNemar’s exact test. An ablation analysis indicated that CPM modulation was the main contributor to performance improvement while data augmentation and the classifier architecture further influenced the overall robustness. **Conclusions**: CPM-XNet provides an annotation-efficient strategy for lesion-annotation-free downstream TB classification in CXR images. The findings support preliminary technical feasibility, although larger, naturally imbalanced, cross-institutional validation is required before clinical deployment can be inferred.

## 1. Introduction

Tuberculosis (TB) remains a leading cause of mortality from infectious diseases worldwide and accounts for millions of new cases and deaths annually [1,2]. Early detection of pulmonary TB is essential for timely treatment and effective disease control. Chest X-ray (CXR) imaging has been widely adopted as a first-line screening tool because of its low cost, rapid acquisition, and broad availability, particularly in high-burden regions [3,4]. However, identifying TB in CXR images is challenging [4]. Manifestations of TB are often subtle, heterogeneous, and overlapping with other pulmonary conditions, and an experienced radiologist is needed for accurate diagnosis. However, many healthcare systems face a shortage of trained specialists and increase in the imaging workload, which has created an urgent need for reliable automated screening tools [5]. Over the past decade, deep-learning-based computer-aided diagnosis (CAD) systems [5,6,7] have seen a rapid evolution and shift from conventional classifiers such as convolutional neural networks (CNNs) to deeper architectures such as ResNet, DenseNet, and EfficientNet evaluated on diverse public datasets. In controlled settings, such systems have achieved a diagnostic performance comparable to that of expert radiologists [6,8]. However, most high-performing CAD systems depend on densely annotated datasets such as pixel-level lung masks or lesion-level bounding boxes [6,8,9], which are costly and impractical to obtain in routine clinical practice.

In real-world hospital archives, CXR images are typically labeled only at the image level, and they are often derived from radiology reports or clinical diagnoses [10]. When trained directly on such weakly labeled data, conventional classifiers are prone to learning spurious correlations outside lung regions (e.g., text markers, pacemakers, or collimator edges). This is a phenomenon known as “shortcut learning,” and it leads to unstable predictions and limited generalizability [11]. To mitigate the risk of learning irrelevant features, previous segmentation-assisted approaches have incorporated lung masks as a preprocessing step [7,12,13]. However, these methods require pixel-level annotations or complex pretrained models, which introduces a potential domain bias [12,13]. While attention-based mechanisms have been explored, attention alone does not guarantee anatomically meaningful focus under weak supervision [14,15]. More recent annotation-efficient strategies including multiple-instance learning, weakly supervised learning, and knowledge distillation have demonstrated promising results [16,17,18,19], but they typically involve complex multistage pipelines or extensive pretraining [20], limiting their practicality for clinical deployment.

This fundamental mismatch between research datasets and clinical reality motivated us to develop CPM-XNet, an annotation-efficient deep-learning framework for lesion-annotation-free downstream TB classification in CXR images. The proposed framework incorporates a compressing–projecting mask (CPM) that provides soft lung-aware modulation without hard cropping to suppress extra-pulmonary confounders while preserving global image context. We systematically evaluated the effects of CPM, data augmentation, classifier architecture, and transfer learning across internal and public datasets to demonstrate how CPM-XNet improves the classification performance and produces anatomically more focused feature responses and to support its potential value as a preliminary, anatomically guided framework for TB classification.

## 2. Materials and Methods

### 2.1. Data

To rigorously evaluate CPM-XNet and ensure its robustness across varying clinical environments, we compiled a heterogeneous dataset from three distinct sources encompassing diverse patient demographics, hardware vendors, and acquisition protocols:

1. **Tung (Internal):** This primary dataset comprised hospital-based frontal CXR images acquired from an institutional picture archiving and communication system in Taiwan. The dataset was strictly labeled at the image level as TB-positive or normal based on a combination of radiologist reports, clinical follow-ups, and microbiological confirmations. No region-of-interest or pixel-level annotations were available, which perfectly reflected the weakly supervised conditions of routine clinical archives. Prior to inclusion, all images were completely anonymized to ensure compliance with Institutional Review Board ethical standards.
2. **TBX11K** [10]: To challenge deep-learning models with large-scale variance, we incorporated the TBX11K public dataset. Although TBX11K contains extensive bounding box annotations for active and latent TB, we intentionally discarded all localization metadata during the training phase. We utilized only the binary image-level labels to strictly adhere to our annotation-efficient paradigm.
3. **Montgomery and Shenzhen (MS)** [9]: The Montgomery dataset was collected from the TB screening program in Montgomery County, Maryland, USA, while the Shenzhen dataset was acquired from Shenzhen No. 3 Hospital in China. These datasets represent critical international benchmarks in pulmonary imaging.

The MS datasets, which included precise and expert-annotated lung boundary masks, were isolated and used exclusively for the initial pretraining of the CPM module. This separation was designed to minimize the risk of data leakage between segmentation prior learning and downstream TB classification tasks. Table 1 details the data partitioning and class distribution.

As summarized in Table 1, the “Train/Validation” columns indicate the data pool used before the final internal split. This pool was randomly divided into training and validation subsets using an 80/20 split. For TBX11K, the proportions were intentionally different from a conventional 70/10/20 or 80/10/10 split. A small class-balanced subset containing 123 TB-positive and 123 normal images was selected as an additional training/validation source, whereas the remaining imbalanced subset, consisting of 3088 TB-positive and 483 normal images, was retained for external testing. This design allowed TBX11K to serve both as a source of training diversity and as an imbalanced external evaluation set.

To reduce information leakage, CPM generator pretraining and downstream TB classifier training were separated. The BN U-Net used in the CPM module was pretrained using lung-field masks from the MS dataset and did not use downstream TB diagnostic labels for classifier optimization. After CPM pretraining, the CPM weights were frozen and used as a lung-aware anatomical modulation component. The downstream classifier was trained using image-level TB/normal labels from the classification training subsets. Held-out test images were not used for classifier training, validation, hyperparameter selection, CPM optimization, or weight updates.

### 2.2. Proposed Framework

**Figure 1:**
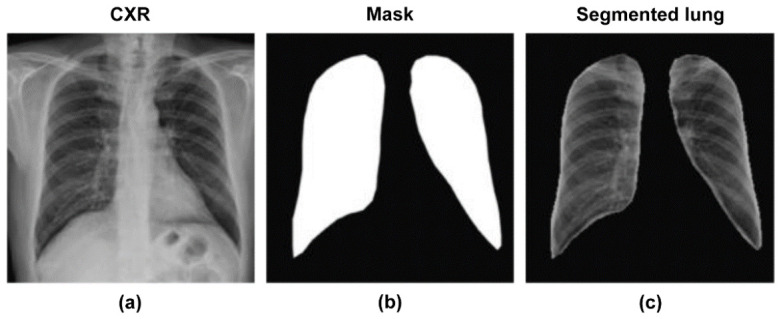
Overview of CPM-XNet. (**a**) Original CXR image input. (**b**) Generation of the compressing–projecting mask (CPM) for soft lung-aware modulation. (**c**) CPM-modulated CXR image used for downstream tuberculosis classification.

CPM-XNet is based on integrating lung-aware feature modulation directly into the classification pipeline. Conventional CNNs trained on whole CXR images often exhibit shortcut learning where the model exploits non-pathological confounders such as laterality markers [11], pacemaker leads, or clavicle bone shadows to optimize the loss function. To counteract this, the CPM module generates a continuous-valued spatial attention map that algorithmically highlights the lung parenchyma. This map is then projected back onto the original raw image to produce a lung-aware and context-preserved representation. Because CPM-XNet does not utilize hard-thresholding, which would blindly assign a value of 0 to background pixels, global contextual information such as pleural effusions that may obscure the standard lung boundary remains intact, as shown in Figure 1.

### 2.3. Compressing–Projecting Mask

#### 2.3.1. Mathematical Formulation

Let the input CXR image be defined as the tensor $X\;\in \;R^{H\;\times \;W\;\times \;C}$, where *H* and *W* denote the spatial dimensions and *C* = 1 for grayscale CXR images. The CPM module, which is parameterized by the weight $\theta_{CPM}$, acts as an anatomical function $f_{CPM}\left(X;\theta_{CPM}\right)$ that outputs the spatial modulation map $M\in {\left[0,1\right]}^{H\times W}$. This map encodes the relative diagnostic importance of each spatial coordinate $\left(i,j\right)$. The projecting operation generates the modulated input tensor $X^{\prime }$ via element-wise (Hadamard) multiplication:

(1) $$X^{\prime }=X⊙\left(1+\alpha M\right),$$

where α is a scaling hyperparameter that determines the intensity of the projection. In the present study, α was fixed at 1 to preserve the original image intensity scale while allowing CPM to act as a soft multiplicative anatomical modulation factor. Alternatively, the pure soft-masking approach can be defined as

(2) $$X^{\prime }=X⊙M.$$

**Figure 2:**
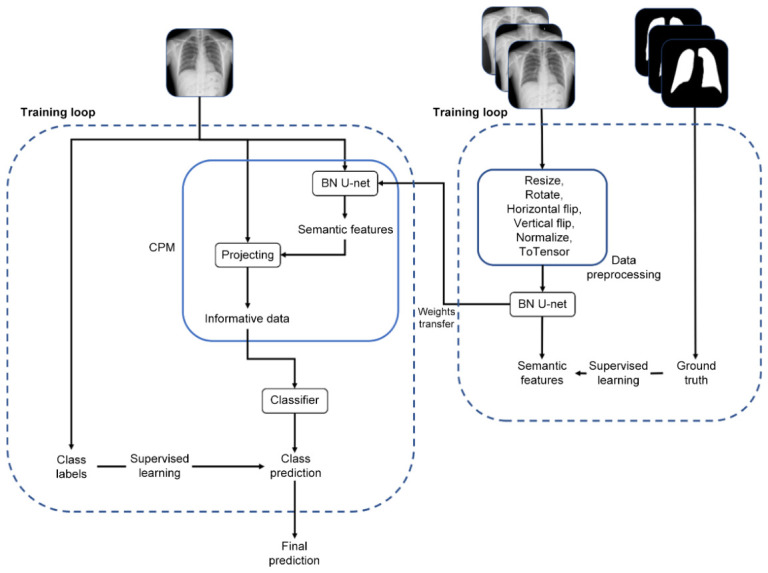
CPM modulation of CXR images.

By utilizing soft continuous values of M with a range of 0–1 rather than a binary threshold M ∈{0, 1}, the CPM module ensures that gradients can flow continuously during backpropagation. As shown in Figure 2, this reduces sensitivity to minor segmentation inaccuracies while effectively compressing extra-pulmonary noise [7,12,13].

#### 2.3.2. Implementation

**Figure 3:**
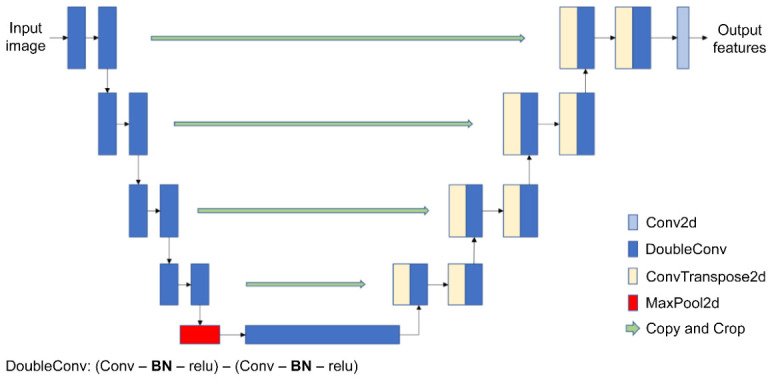
Structure of BN U-Net.

Batch normalization (BN) U-Net was used as the CPM module. It is based on the highly successful U-Net topology [21] and comprises a contracting (encoder) path to capture structural context and a symmetric expanding (decoder) path to enable precise localization. To address the severe internal covariate shift caused by differently calibrated X-ray machines, we strategically inserted BN layers immediately following each 3 × 3 convolution before the rectified linear unit (ReLU) activation. As shown in Figure 3, this transforms each DoubleConv block into a repeating sequence of convolution–BN–ReLU layers. BN stabilizes the latent representations across heterogeneous mini-batches:

(3) $$y^{\left(k\right)}=\gamma^{\left(k\right)}\left(\frac{x^{\left(k\right)}-\mu_{B}}{\sqrt{\sigma_{B}^{2}+\epsilon }}\right)+\beta^{\left(k\right)},$$

where $\mu_{B}$ and $\sigma_{B}^{2}$ are the mini-batch mean and variance, ϵ is a small constant for numerical stability, and γ, β are learnable scale and shift parameters.

**Figure 4:**
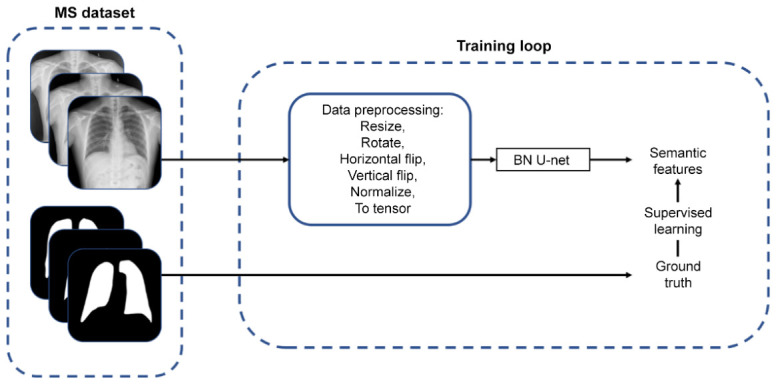
Training process of BN U-Net.

During the pretraining phase on the MS dataset, BN U-Net was trained in a supervised learning manner. Figure 4 illustrates the comprehensive training pipeline, which includes data preprocessing (i.e., resizing, rotating, flipping, normalization) and semantic feature extraction. BN U-Net was optimized by using a Dice coefficient loss to maximize the spatial overlap between the predicted mask and expert ground truth. Once converged, the weights $\theta_{CPM}$ were strictly frozen to serve as an immutable anatomical prior and prevent co-adaptation with the downstream classifier.

### 2.4. Performance Evaluation

#### 2.4.1. Classifier Comparison

**Figure 5:**
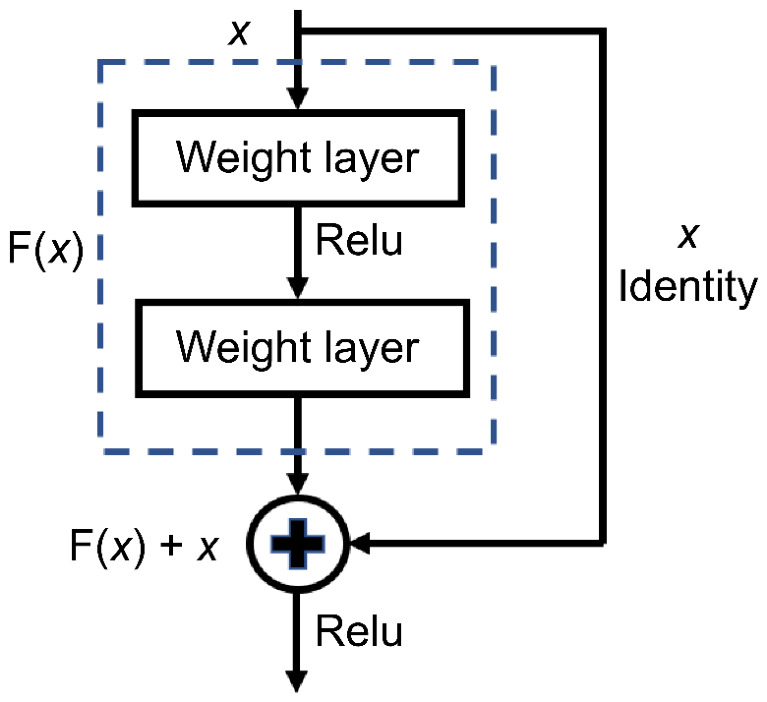
Basic residual block built by residual skip connections.

To comprehensively benchmark the downstream feature extraction capabilities of CPM-XNet, we systematically evaluated the performance of multiple state-of-the-art classifier architectures using the modulated input $X^{\prime }$:

1. ResNet (50, 101, 152) [22]: As shown in Figure 5, these classifiers employ residual skip connections $\left(F\left(x\right)+x\right)$ to alleviate the vanishing gradient problem, which facilitates the training of extremely deep networks.
2. DenseNet-121 [23]: This classifier maximizes information flow by concatenating feature maps from all preceding layers within a dense block, which promotes feature reuse and reducing the total parameter count.
3. EfficientNet [24]: This classifier utilizes a compound scaling heuristic that uniformly scales network width, depth, and resolution to optimize the computational efficiency.
4. Vision Transformer (ViT) [25]: This classifier explores a non-convolutional paradigm by flattening the image into a sequence of 16 × 16 non-overlapping patches and utilizing multi-head self-attention mechanisms to capture global dependencies.

#### 2.4.2. Experimental Settings

All computational experiments were executed on a high-performance deep-learning workstation equipped with an NVIDIA GeForce RTX 3090 graphics processing unit with 24 GB of video random access memory. CPM-XNet and the other classifiers were implemented by using the open-source PyTorch framework (version 1.13.1) in Python (version 3.9). Hardware acceleration was achieved by utilizing CUDA (version 11.7) and cuDNN libraries to ensure optimal computational efficiency and strict experimental reproducibility.

The classifiers were trained end-to-end to minimize the binary cross-entropy loss:

(4) $$L_{BCE}=-\frac{1}{N}\sum_{i=1}^{N}\left[y_{i}\log\left(\hat{y_{i}}\right)+\left(1-y_{i}\right)\log\left(1-\hat{y_{i}}\right)\right],$$

where N is the batch size, $y_{i}\in \{0,1\}$ is the ground-truth image-level label, and $\hat{\;y_{i}}$ is the predicted probability of TB. We employed the Adam optimizer [26] with initial moment estimates $\beta_{1}$ = 0.9 and $\beta_{2}$ = 0.999. The initial learning rate was set to 1 × 10^−4^ and was regulated by using a ReduceLROnPlateau scheduler, which decreased the learning rate by a factor of 0.1 if the validation loss stagnated for five consecutive epochs. An early stopping mechanism with a patience of 15 epochs was implemented to prevent overfitting. Data augmentation was aggressively applied to simulate real-world clinical perturbations [27]. Geometric transformations included random rotations (±15°), horizontal flipping, and minor translations. Intensity-based augmentations included Gaussian blur to simulate motion artifacts and contrast-limited adaptive histogram equalization (CLAHE) to simulate varying penetration levels in radiographic acquisition. We also conducted parallel experiments comparing random initialization (i.e., training from scratch) against transfer learning using weights pretrained on the ImageNet database [20].

#### 2.4.3. Evaluation Metrics

The model efficacy was quantified by using standard statistical measures for medical diagnostic tests: accuracy (ACC), area under the receiver operating characteristic curve (AUC-ROC), sensitivity (Sn), and specificity (Sp). To account for varying degrees of class imbalance inherent in clinical datasets, we heavily emphasized the F1 score (i.e., harmonic mean of precision and sensitivity) and Matthews correlation coefficient (MCC). MCC provides a balanced measure that is only high if the prediction yields good results in all four categories of the confusion matrix (i.e., true positives, false positives, true negatives, false negatives) [9,10]. Furthermore, the 95% confidence intervals (CIs) for the AUC-ROC were analytically estimated by using the Hanley and McNeil method [28] to ensure the statistical reliability of the discriminative performance regardless of the test set sample size.

#### 2.4.4. Repeated-Seed Evaluation and Statistical Analysis

To address the potential instability associated with single-run evaluation in a small medical imaging dataset, the main configuration, ResNet-101 with CPM modulation, was repeated using five different random seeds. The corresponding ResNet-101 model without CPM modulation was also trained and evaluated using the same five random seeds. For each run, the same held-out Tung test cohort was used for evaluation, and all major diagnostic metrics, including accuracy, sensitivity, specificity, F1-score, AUC-ROC, and MCC, were calculated. The repeated-run results are reported as mean ± standard deviation across the five random seeds.

To assess whether the performance improvement associated with CPM modulation was statistically significant at the per-case level, McNemar’s exact test was performed on the Tung cohort. This paired test compared the correctness of CPM-ResNet101 and non-CPM ResNet101 predictions on the same test cases for each random seed. A two-sided *p* value < 0.05 was considered statistically significant.

## 3. Results

### 3.1. Classification Performance

Table 2 summarizes the overall classification performance of BN U-Net compared with the baseline U-Net when trained on raw CXR images. BN U-Net consistently outperformed the original U-Net across all evaluation metrics including AUC-ROC, accuracy, F1 score, and MCC. The simultaneous improvement across complementary metrics indicates a genuine enhancement in discriminative capability rather than threshold-specific optimization, which is consistent with observations reported in prior TB CAD benchmarks [5,6,8].

#### 3.1.1. Effect of the Compressing–Projecting Mask

To evaluate the contribution of CPM, we compared the performance of ResNet-50 trained on raw CXR images with that of ResNet-50 trained on CPM-modulated images. Table 3 presents the results. Without CPM, ResNet-50 showed highly imbalanced behavior with an extreme tradeoff between Sn and Sp and AUC-ROC = 0.5, which suggests poor discrimination between TB-positive and normal cases. The pattern of Sn = 1.0 and Sp = 0.0 in the non-CPM condition indicates a degenerate prediction behavior in which the classifier collapsed toward predicting all samples as TB-positive rather than achieving clinically meaningful sensitivity. With CPM, all major metrics improved including F1 score, Acc, AUC-ROC, MCC, and Sp. These findings indicate that CPM helps constrain feature learning toward anatomically relevant lung regions under weak supervision.

#### 3.1.2. Effect of Data Augmentation

We conducted an ablation study to evaluate the effects of three data augmentation methods on the CPM-XNet performance: rotation, Gaussian blur, and CLAHE. Table 4 summarizes the results. Utilizing all three data augmentation methods simultaneously yielded the best outcome. Rotation produced the most pronounced individual performance gain, which reflects the sensitivity of clinical CXR image acquisition to positional variability. Disabling rotation (i.e., using only CLAHE and Gaussian blur) resulted in a notable drop in AUC-ROC. Meanwhile, Gaussian blur and CLAHE enhanced robustness by simulating image quality degradation and contrast variation.

To strengthen data diversity, we mixed the Tung dataset with a subset of the TBX11K dataset to form the Mix dataset. We then applied the three data augmentation methods again to evaluate the effects on the performance of CPM-XNet. As presented in Table 5, introducing external mixed data improved all metrics. With the addition of data augmentation, CPM-XNet achieved an outstanding AUC-ROC of 0.9167 (95% CI: 0.86–0.97).

Combining all data augmentation methods achieved the best overall performance, which indicates that they had complementary rather than redundant effects. Table 6 presents the detailed performance metrics of all classifiers using the augmented Mix dataset.

#### 3.1.3. Repeated-Seed Evaluation of ResNet-101 with and Without CPM

To further evaluate the robustness of the main configuration, ResNet-101 with CPM modulation was repeated using five random seeds and compared with the corresponding ResNet-101 model without CPM modulation. The five-seed mean ± standard deviation results are summarized in Table 7. Across five repeated runs, CPM-ResNet101 achieved an accuracy of 89.8% ± 4.1%, AUC-ROC of 0.944 ± 0.012, sensitivity of 91.7% ± 9.5%, specificity of 88.0% ± 1.8%, F1-score of 89.8% ± 4.9%, and MCC of 0.801 ± 0.081. In contrast, ResNet-101 without CPM showed markedly lower and unstable performance, with an accuracy of 49.3% ± 1.5%, AUC-ROC of 0.500 ± 0.001, sensitivity of 4.3% ± 9.7%, specificity of 94.3% ± 12.7%, F1-score of 5.8% ± 12.9%, and MCC of −0.015 ± 0.034.

A paired McNemar’s exact test was performed on the Tung cohort to compare CPM-ResNet101 and non-CPM ResNet101 using the same test cases. CPM-ResNet101 showed significantly better paired classification performance than the non-CPM model across all five random seeds, with *p* < 0.001 for each comparison. These results indicate that the improvement associated with CPM modulation was unlikely to be explained solely by random variation in the test set. Because McNemar’s exact test was based on per-case paired correctness rather than aggregate performance metrics, this analysis directly addressed whether CPM changed the classification outcomes on the same Tung test cases.

#### 3.1.4. Effect of the Classifier Architecture

CNN-based classifiers consistently outperformed the transformer-based ViT under the experimental settings. Deeper residual networks achieved the highest AUC-ROC and MCC values, which indicates that CPM-modulated inputs were more effectively utilized by architectures with a convolutional inductive bias. In contrast, ViT showed a limited performance under annotation-limited and data-constrained conditions, which is consistent with prior reports that transformer-based models may require substantially larger training datasets to fully realize their advantages in medical imaging tasks [20,25]. The near-random or single-class prediction patterns observed in VGG19 and ViT, particularly ACC = 0.5 and MCC = 0, should be interpreted as classifier collapse or unstable optimization under limited training data rather than meaningful diagnostic performance. Table 8 summarizes the ViT results when trained from scratch and when utilizing transfer learning.

#### 3.1.5. Effect of Transfer Learning

The effect of transfer learning was architecture-dependent. For some models, ImageNet-pretrained weights provided modest improvements. However, deeper residual networks still performed strongly when trained from scratch. This suggests that the usefulness of transfer learning may depend on the interaction between the backbone architecture, dataset scale, and CPM. Table 9 presents the complete experimental results for all classifiers.

### 3.2. External Validation

An external validation demonstrated that CPM-XNet maintained balanced sensitivity and specificity across datasets acquired from different sources. The limited performance degradation observed across datasets suggests that lung-aware feature modulation reduces sensitivity to dataset-specific biases, which circumvents the shortcut learning often observed in artificial intelligence applications [11] and is consistent with findings from prior multi-institutional TB screening studies [29]. To further contextualize the contributions of this study, Table 10 summarizes previously reported deep-learning applications for TB-related CXR classification. This comparison should be interpreted strictly as descriptive and contextual rather than as a direct head-to-head benchmark. Direct numerical ranking is inappropriate because the listed studies differ substantially in dataset composition, sample size, TB case definition, annotation availability, preprocessing strategy, model architecture, and validation protocol. Therefore, Table 10 is retained to place CPM-XNet within the broader TB-related CXR classification literature, not to establish superiority over prior methods.

### 3.3. Qualitative Analysis

Representative qualitative visual analysis suggested that CPM may encourage more anatomically coherent and lung-centered feature responses [15]. In contrast, classifiers without CPM appeared to exhibit more diffuse and inconsistent attention patterns, which may reflect reliance on non-lung or shortcut-related features. However, these visual findings should be interpreted as qualitative observations rather than definitive localization evidence. By providing intuitive visual representations, CPM-XNet may help reviewers assess whether the model response is located within clinically relevant lung regions, which remains important for interpretability in high-stakes clinical AI applications [31]. Future studies should incorporate quantitative localization metrics, such as lung activation ratio, saliency overlap with lung masks, IoU-based analysis, or pointing-game accuracy, to more objectively evaluate anatomical focus.

## 4. Discussion

Our results suggest that CPM-XNet addresses a practical limitation of deep-learning models applied to weakly supervised TB classification: a tendency to rely on non-lung cues when trained directly on whole-image labels [11]. During unsupervised training, classifiers may exploit image markers, device-related artifacts, or peripheral background patterns that correlate with class labels but are not directly related to pulmonary pathology. By introducing CPM as a soft anatomical prior, the proposed framework suppresses irrelevant background responses and encourages feature extraction within the lung fields [7,12,13]. Because the modulation is continuous rather than binary, potentially informative contextual regions are not completely removed. This design may be particularly important for cases with diffuse disease extents or boundary-adjacent abnormalities.

The repeated-seed analysis further supports the robustness of CPM modulation under limited-data conditions. Although CPM seed 1 showed lower sensitivity than the other CPM seeds, it was retained in the primary analysis because there was no evidence of technical failure, data mismatch, or a predefined exclusion criterion that would justify its removal. Removing this seed post hoc would risk selective reporting and underestimate seed-dependent variability. Importantly, even the lowest-performing CPM seed substantially outperformed the corresponding non-CPM model. In the non-CPM condition, four of five seeds collapsed into predicting all test cases as normal, resulting in zero sensitivity. This finding suggests that CPM modulation not only improved average classification performance but also helped stabilize ResNet-101 training across different random initializations.

We observed a profound disparity in efficacy between convolution-based and transformer-based classifiers within our specific data regime. ResNet-101 achieved the highest performance in the present experiments, with an MCC of 0.9005. Conversely, ViT struggled greatly with its performance, effectively near random chance when trained from scratch and only achieving a modest performance after ImageNet transfer learning [20]. This divergence suggests the value of convolutional inductive biases such as translation equivariance and locality in medical imaging tasks characterized by relatively limited sample sizes. ViT treats images as sequences of patches and must independently learn the spatial relationships between them. These findings are consistent with prior reports that transformer-based models often require substantially larger datasets and that transfer learning from natural images may not always be optimal for medical imaging tasks [20,25].

Interpretability remains an important consideration for the clinical deployment of deep-learning models. The qualitative analysis suggested that CPM led to a greater concentration on lung feature responses than unguided classification. Although this does not by itself establish full clinical explainability, it indicates that soft lung-aware modulation may improve the transparency of model behavior by reducing attention to extra-pulmonary artifacts. Such an anatomically coherent response may make model outputs easier to interpret during clinical review [15].

Automated CXR interpretation extends beyond binary TB classification because thoracic radiographs may contain overlapping or coexisting abnormalities. Kufel et al. investigated transfer learning-based multi-label classification of 14 CXR abnormalities using the NIH ChestX-ray14 dataset, highlighting the broader complexity of deep-learning-based CXR interpretation [32]. This context is relevant to TB classification because TB-related findings may overlap with other thoracic abnormalities such as consolidation, fibrosis, pleural abnormality, nodular opacity, and infiltrative changes. Therefore, future studies should evaluate whether CPM-based anatomical modulation remains useful in multi-label or multi-pathology CXR interpretation settings.

Several limitations should be acknowledged. First, the dataset mainly comprised frontal CXR images from adults, and CPM-XNet was not specifically evaluated for pediatric TB or atypical radiographic presentations. Second, CPM-XNet was developed for image-based TB classification and cannot distinguish active disease from prior healed lesions without additional clinical information. Third, although external validation showed encouraging generalizability, broader multi-center testing is still required to confirm its robustness across institutions, scanners, and prevalence settings. Future work may involve extending CPM-XNet by incorporating clinical metadata, lighter backbones for edge deployment, and prospective evaluation in workflow-oriented screening settings. In addition, the Tung internal dataset was relatively small and was balanced by design; therefore, the present results should be interpreted as preliminary technical validation rather than evidence of clinical deployment readiness in naturally imbalanced screening populations. CPM-XNet should also not be interpreted as fully annotation-free in an absolute sense, because the CPM generator relies on lung-field annotations during pretraining; its annotation efficiency refers specifically to downstream TB classification, for which no TB lesion bounding boxes, lesion masks, or lesion-level region annotations are required. Finally, although repeated-seed evaluation and paired McNemar’s exact testing were added for the main ResNet-101 CPM comparison on the Tung cohort, comprehensive statistical comparisons across all model architectures, quantitative localization metrics for visual explanations, and systematic computational profiling were not performed and should be addressed in future work.

## 5. Conclusions

CPM-XNet is an annotation-efficient deep-learning framework for lesion-annotation-free downstream TB classification in CXR images. By embedding lung-aware feature modulation into the classification pipeline, the proposed framework demonstrated promising technical feasibility using image-level TB/normal labels for downstream classifier training. Repeated-seed evaluation and paired testing on the Tung cohort further supported the robustness of CPM modulation in the main ResNet-101 configuration. However, CPM-XNet should not be interpreted as fully annotation-free in an absolute sense, because the CPM generator relies on lung-field annotations during pretraining. Further validation in larger, naturally imbalanced, cross-institutional cohorts is required before clinical deployment can be inferred.

## Figures and Tables

**Table 1:** Composition and partitioning of datasets used in this study.

Dataset	Train/Validation Positive	Train/Validation Negative	Test Positive	Test Negative
Tung	247	247	60	60
TBX11K	123	123	3088	483
MS	654	654	50	50

**Note.** The “Train/Validation” columns indicate the data pool used before the internal 80/20 training–validation split.

**Table 2:** Downstream accuracy and F1 score of U-Net and BN U-Net trained on raw CXR images.

Index	U-Net	BN U-Net
Accuracy	0.9669	0.9714
F1 Score	0.9298	0.9406

**Table 3:** Performance comparison of ResNet-50 trained with and without CPM.

Index	Without CPM	With CPM
F1 Score	0.3333	0.7333
ACC	0.5	0.7333
AUC-ROC	0.5	0.7333
MCC	0	0.4667
Sn	1	0.7
Sp	0	0.7667

**Table 4:** Effects of data augmentation strategies on CPM-XNet performance.

Index	Without Augmentation	All 3 Methods	CLAHE + Rotation	Gaussian Blur + Rotation	CLAHE + Gaussian Blur
F1 Score	0.733	0.8082	0.7499	0.7913	0.6999
MCC	0.4667	0.6174	0.5003	0.5854	0.4002
AUC-ROC	0.7333	0.8083	0.75	0.7917	0.7
ACC	0.7333	0.8083	0.75	0.7917	0.7
Sn	0.7188	0.8246	0.7719	0.8181	0.6935
Sp	0.75	0.7937	0.7419	0.7692	0.7069

**Table 5:** Performance improvement using Mix dataset and augmentation.

Index	Original Dataset	Mix Dataset	Mix Dataset with Augmentation
F1 Score	0.733	0.8326	0.9165
MCC	0.4667	0.6727	0.8375
AUC-ROC	0.7333	0.8333	0.9167
ACC	0.7333	0.8333	0.9167
Sn	0.7188	0.8846	0.963
Sp	0.75	0.7941	0.8787

**Table 6:** CPM-XNet performance with different classifiers trained on the augmented Mix dataset.

Classifier	Sn	Sp	F1 Score	ACC	AUC-ROC (95% CI)	MCC
VGG19	0	1	0	0.5	0.5	0
ResNet-50	0.9667	0.8667	0.9165	0.9167	0.9167 (0.86–0.97)	0.8375
ResNet-101	0.9667	0.9355	0.95	0.95	0.9500 (0.91–0.99)	0.9005
ResNet-152	0.9649	0.9206	0.9416	0.9417	0.9417 (0.89–0.98)	0.8844
DenseNet-121	0.9667	0.8833	0.9249	0.925	0.9250 (0.87–0.97)	0.853
EfficientNet	0.9333	0.9167	0.925	0.925	0.9250 (0.87–0.97)	0.8501
ViT	0	1	0	0.5	0.5	0

**Table 7:** Five-seed repeated evaluation of threshold-dependent metrics for ResNet-101 with and without CPM on the Tung cohort.

Model	Seeds	Accuracy	Sensitivity	Specificity	F1-Score	MCC
ResNet-101 without CPM	5	49.3 ± 1.5	4.3 ± 9.7	94.3 ± 12.7	5.8 ± 12.9	−0.015 ± 0.034
ResNet-101 with CPM	5	89.8 ± 4.1	91.7 ± 9.5	88.0 ± 1.8	89.8 ± 4.9	0.801 ± 0.081

**Table 8:** Effects of the training mode on ViT.

Mode	Sn	Sp	F1 Score	ACC	AUC-ROC	MCC
Sc	0	1	0	0.5	0.5	0
Tr	0.8	0.6333	0.7147	0.7167	0.7167	0.4395

Sc: trained from scratch; Tr: transfer learning with ImageNet weights.

**Table 9:** Effects of the training mode on all classifiers.

Classifiers	Mode	Sn	Sp	F1 Score	ACC	AUC-ROC	MCC
VGG19	Sc	0	1	0	0.5	0.5	0
VGG19	Tr	0	1	0	0.5	0.5	0
ResNet-50	Sc	0.9667	0.8667	0.9165	0.9167	0.9167	0.8375
ResNet-50	Tr	0.9333	0.9333	0.9333	0.9333	0.9333	0.8667
ResNet-101	Sc	0.9667	0.9355	0.95	0.95	0.95	0.9005
ResNet-101	Tr	0.9153	0.9016	0.9083	0.9082	0.9083	0.8667
ResNet-152	Sc	0.9649	0.9206	0.9416	0.9417	0.9417	0.8844
ResNet-152	Tr	0.9286	0.875	0.8999	0.9	0.9	0.8018
DenseNet-121	Sc	0.9667	0.8833	0.9249	0.925	0.925	0.853
DenseNet-121	Tr	0.95	0.9	0.925	0.925	0.925	0.8511
EfficientNet	Sc	0.9333	0.9167	0.925	0.925	0.925	0.8501
EfficientNet	Tr	0.9667	0.9167	0.9416	0.9417	0.9417	0.8501
ViT	Sc	0	1	0	0.5	0.5	0
ViT	Tr	0.8	0.6333	0.7147	0.7167	0.7167	0.4395

Sc: trained from scratch; Tr: transfer learning.

**Table 10:** Comparison of related works.

Author	Year	Database	Number of TB Images	Method	Evaluation Metric
Lakhani et al. [6]	2017	Montgomery County (MC), Shenzhen, Belarus, Thomas Jefferson University Hospital	1007	DCNN ensemble learning, Radiologist	AUC-ROC 0.99
Rahman et al. [7]	2020	Kaggle (for U-Net), NLM, Belarus, NIAID TB Dataset, RSNA CXR Dataset	704 (for U-Net), 7000	Augmented U-Net, 9 pretrained CNNs	ACC 0.986, Sn 0.9856, Sp 0.9854
Park [18]	2022	NIH, BIMCV, CheXpert, India, Montgomery, Shenzhen, Belarus, PADChest, TBX11K	35,985	DISTIL (2 ViTs)	AUC-ROC 0.966
Mohan et al. [30].	2022	Tuberculosis (TB) Chest X-ray Database	7000	Machine learning and VGG19, Feature concatenation	ACC 0.9862
Pasa et al. [29]	2019	MC, Shenzhen, Belarus	1111	Customized CNN	AUC-ROC: 0.811 for MC, 0.9 for Shenzhen, 0.925 for combined
This study	2026	MS (for BN-U-Net), Tung dataset, TBX11K	1308 (MS), 614 (Tung), 3817 (TBX11K)	CPM-XNet	AUC-ROC 0.95, ACC 0.95, Sn 0.9667, Sp 0.9355

## Data Availability

The internal hospital dataset used in this study is not publicly available due to patient privacy and institutional restrictions. De-identified data may be available from the corresponding authors upon reasonable request and with permission from the relevant institution. Publicly available datasets analyzed in this study include TBX11K and the MS chest X-ray datasets.

## Abbreviations

The following abbreviations are used in this manuscript:

TB	Tuberculosis
CXR	Chest X-ray
CAD	Computer-aided diagnosis
CNN	Convolutional neural network
CPM	Compressing–projecting mask
MS	Montgomery and Shenzhen
BN	Batch normalization
ReLU	Rectified linear unit
ViT	Vision Transformer
CLAHE	Contrast-limited adaptive histogram equalization
ACC	Accuracy
AUC-ROC	Area under the receiver operating characteristic curve
Sn	Sensitivity
Sp	Specificity
MCC	Matthews correlation coefficient
CI	Confidence interval
